# Microbial Community Structure and Arsenic Biogeochemistry in Two Arsenic-Impacted Aquifers in Bangladesh

**DOI:** 10.1128/mBio.01326-17

**Published:** 2017-11-28

**Authors:** Edwin T. Gnanaprakasam, Jonathan R. Lloyd, Christopher Boothman, Kazi Matin Ahmed, Imtiaz Choudhury, Benjamin C. Bostick, Alexander van Geen, Brian J. Mailloux

**Affiliations:** a Environmental Science Department, Barnard College, New York, New York, USA; b School of Earth and Environmental Sciences and Williamson Research Centre for Molecular Environmental Science, the University of Manchester, Manchester, United Kingdom; c Department of Geology, University of Dhaka, Dhaka, Bangladesh; d Lamont-Doherty Earth Observatory, Columbia University, Palisades, New York, USA; University of Tuebingen; California Institute of Technology

**Keywords:** arsenic, biogeochemistry, geomicrobiology, metagenomics

## Abstract

Long-term exposure to trace levels of arsenic (As) in shallow groundwater used for drinking and irrigation puts millions of people at risk of chronic disease. Although microbial processes are implicated in mobilizing arsenic from aquifer sediments into groundwater, the precise mechanism remains ambiguous. The goal of this work was to target, for the first time, a comprehensive suite of state-of-the-art molecular techniques in order to better constrain the relationship between indigenous microbial communities and the iron and arsenic mineral phases present in sediments at two well-characterized arsenic-impacted aquifers in Bangladesh. At both sites, arsenate [As(V)] was the major species of As present in sediments at depths with low aqueous As concentrations, while most sediment As was arsenite [As(III)] at depths with elevated aqueous As concentrations. This is consistent with a role for the microbial As(V) reduction in mobilizing arsenic. 16S rRNA gene analysis indicates that the arsenic-rich sediments were colonized by diverse bacterial communities implicated in both dissimilatory Fe(III) and As(V) reduction, while the correlation analyses involved phylogenetic groups not normally associated with As mobilization. Findings suggest that direct As redox transformations are central to arsenic fate and transport and that there is a residual reactive pool of both As(V) and Fe(III) in deeper sediments that could be released by microbial respiration in response to hydrologic perturbation, such as increased groundwater pumping that introduces reactive organic carbon to depth.

## IMPORTANCE

The consumption of arsenic in waters collected from tube wells threatens the lives of millions worldwide and is particularly acute in the floodplains and deltas of southern Asia. The cause of arsenic mobilization from natural sediments within these aquifers to groundwater is complex, with recent studies suggesting that sediment-dwelling microorganisms may be the cause. In the absence of oxygen at depth, specialist bacteria are thought able to use metals within the sediments to support their metabolism. Via these processes, arsenic-contaminated iron minerals are transformed, resulting in the release of arsenic into the aquifer waters. Focusing on a field site in Bangladesh, a comprehensive, multidisciplinary study using state-of-the-art geological and microbiological techniques has helped better understand the microbes that are present naturally in a high-arsenic aquifer and how they may transform the chemistry of the sediment to potentially lethal effect.

## INTRODUCTION

Arsenic (As), is a major threat to the lives of millions of people whose primary water source for drinking and farming is constituted by arsenic-contaminated aquifers in Bangladesh (1, 2). Although the World Health Organization (WHO) guideline value for arsenic in water is 10 µg liter^−1^, the arsenic concentrations reported in Bangladesh aquifers range from <0.5 to 2,500 µg liter^−1^ (3, 4). Previous studies have also shown that arsenite [As(III)] accounts for 90% of the soluble arsenic concentrations in groundwater, whereas arsenate [As(V)] constitutes only 10% (5, 6), and in sediments, the ratio of As(V) to As(III) varies as a function of the mineralogical properties and microbial activities (6–9). Thus, there is a consensus that arsenic is naturally released from sediments to water under microbially induced reducing conditions within the aquifer sediments (10–12). Furthermore, previous studies suggest that the biogeochemical cycling of iron and arsenic, specifically the reduction of mineral assemblages containing Fe(III) and sorbed arsenic(V), plays a critical role in the mobilization of arsenic in aquifers (13–18). Although most of these studies have been based on “lab microcosm” incubations, the precise mechanisms of arsenic release *in situ* remain poorly constrained. In order to understand this complex process in aquifers, it is necessary to correlate various factors, including the quantity of arsenic both in the sediment and in water, the relative abundance of arsenic and the iron speciation and mineralogy in both phases, as well as the relevant microbial communities associated with arsenic hot spots. The aim of this multidisciplinary study is to address the paucity of data available that directly link microbial ecology to the *in situ* geochemistry of arsenic-impacted aquifers, thereby identifying the dominant biogeochemical processes driving arsenic mobilization.

Microbial arsenic reduction occurs through a variety of pathways. Soluble As(V) can be reduced directly to As(III) by microbes during intracellular detoxification processes or can be used to conserve energy for growth via dissimilatory As(V) reduction (19–21). The “AB” gene cluster in bacteria, containing the *ars* and *arr* genes, is used for these detoxification and energy-conserving processes, respectively. During detoxification, the intracellular reduction of As(V) mediated by the ArsC protein is a prerequisite to the efficient export of As(III) from the cell (22, 23). In the case of the dissimilatory arsenic reduction, As(V) is used as the terminal electron acceptor under anoxic conditions, mediated by the terminal arsenate reductase Arr, a molybdoprotein located in the periplasm of Gram-negative bacterial cells. Both of these processes produce As(III), which is usually the dominant form of aqueous arsenic in contaminated aquifers (24–26). Although a wide range of organisms carry the arsenic resistance operon, including many that are not implicated in arsenic mobilization, a narrow distribution of organisms can respire As(V) (27–29). Laboratory incubations (or “microcosms”) using sediments supplied with the addition of ^13^C-labeled carbon sources have also suggested that the expression of *arrA* could be an important factor in controlling the high concentrations of arsenic in aquifers (14, 30–33). However, only a few studies have examined arsenic reduction by microorganisms in the aquifers with geogenic arsenic via the direct analysis of the field samples, which lack exogenous carbon sources and exhibit lower rates of metabolism (34, 35).

The dissimilatory reduction of Fe(III) to Fe(II) can also be energetically favorable for specialist anaerobic microorganisms, including *Geobacter* species, and can result in the solubilization of Fe(II) and/or transformations in the sediment Fe minerology (36, 37). Generally, Fe(III) minerals have more sorption sites for As(III) and As(V) than Fe(II)-bearing minerals. Under reducing conditions, where Fe(III) minerals are dissolved, Fe^2+^ is produced, and Fe(II)-bearing minerals form, the total number of sorption sites for all iron minerals present is expected to be lower (and thus more As is expected to remain in solution). These sorption sites also favor As(V) binding, which can result in increased levels of As(III) in solution (38–40). Sediment extractions show that iron and arsenic are widely correlated in aquifers, indicating that Fe-(hydr)oxides play a critical role in controlling arsenic solubility (41). Extractions with phosphate that target weakly bound arsenic fractions have shown that this surface-bound fraction may be the critical pool of arsenic that governs arsenic mobility, and much of this arsenic may be associated with specific iron minerals. Recent improvements have focused on understanding the relationship between Fe and arsenic speciation using sequential extractions (42, 43), but interpreting such results is often difficult. Other techniques, such as X-ray absorption spectroscopy (XAS), are useful to study this relationship, but have been applied to only a few aquifer materials and even fewer that are systematically related in time and space (37, 44, 45). To date, and to the best of our knowledge, only one study (34) has measured arsenic and Fe speciation in aquifer sediments alongside a preliminary characterization of the extant sediment microorganisms. This information is needed to help determine the roles of the arsenic and Fe redox processes in controlling aquifer arsenic levels *in situ*, and given the improvements in the techniques available for geomicrobiological studies since this initial 2005 paper, the work described in this communication is timely.

Other redox processes could also affect aqueous arsenic levels. For example, As(III) and Fe(II) are reoxidized in the presence of nitrate or oxygen, producing As(V)-sorbing particulate hydrous Fe(III)oxides and oxyhydroxides (46, 47). Similarly, microbial sulfate reduction may influence arsenic solubility through the formation of insoluble arsenic sulfides: e.g., As_2_S_3_ (47–49).

In this study, we hypothesized that if arsenate reduction is the only process leading to arsenic release, the sediment will remain rich in As(V) and the groundwater will contain As(III) that is mobilized during reduction. If Fe(III) reduction alone is responsible for the release of arsenic, then once again the sediment will be dominated by As(V), with As(V) dominant in the groundwater. If both the reduction of As(V) and subsequent sorption to reactive Fe minerals are important for controlling arsenic mobilization, then the sediment will accumulate As(III) until the reactive Fe(III) minerals are reduced and the sorption sites for As(III) are removed. In this scenario, the sediment will become enriched in As(III). Key to differentiating these processes is sampling mineralogy and aqueous levels in the environment at a sufficiently high resolution in order to observe gradients in As and Fe speciation and correlating metal speciation with microbiological data.

The objective of this cross-disciplinary work was, for the first time, to use a broad range of cutting edge molecular techniques in order to (i) determine the speciation of arsenic and iron with depth in the shallow aquifer sediments collected from Bangladesh across an arsenic gradient, (ii) analyze the geochemistry of these two sites, and (iii) compare arsenic speciation with the composition of the bacterial communities, including locating zones where arsenic-respiring bacteria are present, and identifying their potential role in controlling arsenic solubility. This study was conducted in three parts. First, we examined the hydrogeologies of the sediments and water samples from two contrasting sites (sites F and B), followed by analyses of the composition of bacterial communities (and key functional genes that could impact the As speciation). Finally we explored correlations between the microbial community compositions and the data obtained from the hydrogeological analyses of the sediment cores and relevant water samples. This study therefore illustrates the complex interplay between hydrology, mineralogy, and microbiology that underpins arsenic release in aquifer sediments. This work is important, as a more complete understanding of these processes could contribute to more accurate predictions and inform mitigation procedures, for example, linked to interventions that may stimulate or suppress key microbial processes that immobilize or mobilize arsenic into groundwaters.

## RESULTS

**FIG 1:**
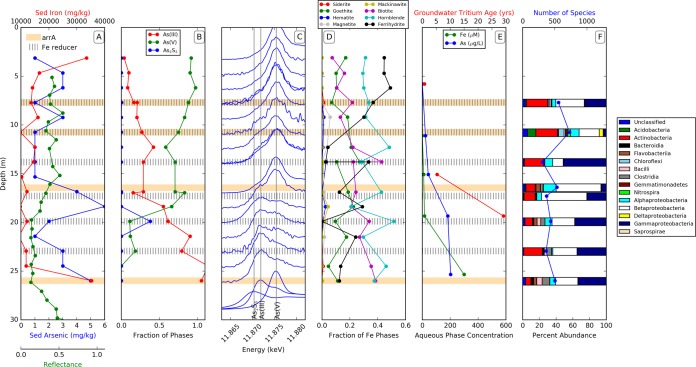
Chemical and microbial ecology analysis of sediments and water from the site F aquifer. (A) Sediment (Sed) arsenic and iron concentration and reflectance curve (blue, red, and green) along the sediment depths with the presence of dissimilatory arsenic-respiring bacteria (brown) and iron-reducing members of the *Geobacteraceae* (lines). (B) As(V), As(III), and AS_2_S_3_ species in the sediment (XANES) at site F. (C) Arsenic X-ray absorption near the edge structure (XANES) spectra along various depths of sediments. (D) Fraction of the Fe phases in relation to the Fe minerals along various depths. (E) As, Fe concentration (blue and green) in wells (aqueous phase), and groundwater tritium age (red). (F) 16S rRNA gene analysis for the microbial communities. Bar diagrams indicate the percentage of each class of the bacterial kingdom.

**FIG 2:**
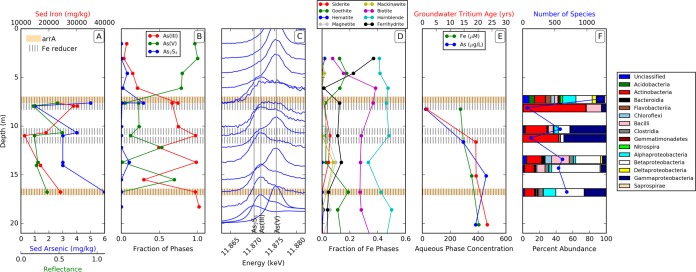
Chemical and microbial ecology analysis of sediments and water from the site B aquifers. (A) Sediment (Sed) arsenic and iron concentration and reflectance curve (blue, red, and green) along the sediment depths with the presence of dissimilatory arsenic-reducing bacteria (brown) and iron-reducing members of the *Geobacteraceae* (lines). (B) As(V), As(III), and AS_2_S_3_ species in the sediment (XANES) at site B. (C) Arsenic X-ray absorption near the edge structure (XANES) spectra along various depths of sediments. (D) Fraction of Fe phases in relation to the Fe minerals along various depths. (E) As, Fe concentration (blue and green) in wells (aqueous phase), and groundwater tritium age (red). (F) 16S rRNA gene analysis for the microbial communities. The bar diagram indicates the percentage of each class of the bacterial kingdom.

Key data are presented in Fig.  1 and 2 in order to provide a synopsis of the contrasting biogeochemistries of the two sample sites and their associated microbiology. Tables S1 and S2 in the supplemental material show the comprehensive data from the various mineralogical and molecular investigations conducted on samples from sites F and B in this study.

10.1128/mBio.01326-17.8 TABLE S1  Chemical and molecular ecology analysis of sediments and water from the site F aquifer. Download TABLE S1, DOCX file, 0.1 MB. Copyright © 2017 Gnanaprakasam et al. 2017 Gnanaprakasam et al. This content is distributed under the terms of the Creative Commons Attribution 4.0 International license.

10.1128/mBio.01326-17.9 TABLE S2  Chemical and molecular ecology analysis of sediments and water from the site B aquifer. Download TABLE S2, DOCX file, 0.1 MB. Copyright © 2017 Gnanaprakasam et al. 2017 Gnanaprakasam et al. This content is distributed under the terms of the Creative Commons Attribution 4.0 International license.

### Site F. (i) Sediment chemistry.

Site F is uniformly sand from approximately 30 m below the ground surface (bgs) to the surface and is characterized by a relatively rapid recharge rate of 0.5 m year^−1^. The arsenic concentration of the 15 sediment samples was 2 to 3 mg kg^−1^, with some values as low as 1 and as high as 6 mg kg^−1^ at a depth of 18.4 m (Fig. 1A). The variation in arsenic levels mirrored changes in solid-phase Fe and Mn. The Fe concentrations in the sediments were highest at the surface and at depth but were typically 10 to 20 g kg^−1^ and ranged from 9.3 to 35.3 g kg^−1^. Manganese concentrations followed a similar trend to Fe in relation to depth, with the lowest concentration, 174 mg kg^−1^, observed at 16.9 m bgs and the highest concentration, 753 mg kg^−1^, observed at a depth of 26 m bgs.

Solid-phase Fe and arsenic speciation was determined at 17 depths ranging from 3.1 to 26 m bgs. At site F, arsenic speciation changed smoothly as a function of depth. From the surface to 18.4 m bgs, the solid-phase arsenic gradually decreased from almost entirely As(V) to 58% As(V) at 12.3 m bgs. From 19.9 to 26 m bgs, the percentage of As(V) rapidly decreased, with appreciable As_2_S_3_ found only in this transitional zone, reaching 38% of the total arsenic at 19.9 m bgs. Below 19.9 m bgs, the sediment was dominated by As(III) with less than 20% As(V) (Fig. 1B). The iron mineralogy of authigenic Fe minerals also changed gradually as a function of depth. At the surface, most Fe was present as a combination of ferrihydrite and goethite, with similar quantities of Fe in silicates. Above 10 m, ferrihydrite represented as much as 50% of the total Fe and decreased with depth. Goethite consistently represented 20 to 30% of the total Fe at all depths. The Fe(II, III) silicates, modeled with biotite and hornblende, were widely variable but showed no trends with depth and together represented about half of the total Fe. By 30 m bgs, the abundance of Fe(III) decreased, particularly when only considering the abundance of nonsilicate Fe species, and Fe(II)-bearing minerals were detected, although they were still relatively minor species (Fig. 1D).

### (ii) Aqueous chemistry.

The aqueous chemistry of 5 well-samples from site F showed the presence of arsenic ranging from 0.5 µg liter^−1^ at shallow depths to 203 µg liter^−1^ at 26 m bgs, far in excess of the 10 µg liter^−1^ from the WHO guideline. The pH of the water samples ranged between 6.3 and 7.0, and the dissolved oxygen concentration remained below the levels of detection, providing suitable conditions for the neutrophilic anaerobic metal-reducing bacteria implicated in arsenic mobilization. The soluble Fe concentrations paralleled these changes, increasing from 0.008 mg liter^−1^ (0.15 µM) near the surface to 17 mg liter^−1^ (300 µM) at 26 m bgs. There was a clear relationship between soluble arsenic and Fe concentrations in the wells of each depth (Fig. 1E). In contrast, the sulfur concentrations in the wells decreased with depth, from 2.1 mg liter^−1^ (65 µM) at 6.2 m to 0.13 mg liter^−1^ (4.2 µM) at 26 m.

### Microbial ecology. (i) 16S rRNA gene-based community analysis.

A total of 70,907 16S rRNA gene sequences were obtained for site F by processing pyrosequencing data through the QIIME pipeline. The number of sequences obtained for each depth varied from 5,788 to 10,426, and the alpha rarefaction analyses suggested that the number of distinct species detected at each depth ranged between 308 at 16.9 m and 729 at 10.8 m (Fig. 1F).

All sediment intervals except 10.8 and 16.9 m were dominated by an organism most closely related to *Acinetobacter* sp. strain TTH0-4 (99.0% identity) (50). *Acinetobacter* species are normally associated with oxic environments, being nonfermentative, aerobic bacteria that decompose large organic molecules (51), although use of stored polyphosphate as an intracellular energy source can support metabolism in environments undergoing redox transitions (52). Although *Acinetobacter* species have been detected previously in arsenic-contaminated sites in Assam (53) and the Hubei Province in China (54), they have not been recognized as As(V) [or indeed Fe(III)]-respiring bacteria, even if *Acinetobacter* sp. strain TTH0-4 is known to carry the genes coding for arsenic resistance proteins (ArsC and ArsH). Of the total community composition, a close relative of *Acinetobacter* sp. strain TTH0-4 comprised 21% of the bacterial population at a depth of 7.7 m, 11% at 13.8 m, 4% at 16.9 m, 11% at 19.9 m, 27% at 22.9 m, and 11% at 26 m. Other dominant species found in almost all depths included organisms most closely related to known nitrate-reducing bacteria, such as *Massilia brevitalea* (98.4% identity) (55), *Psychrobacter glacincola* (97.4% identity) (56), and *Arthrobacter defluvii* (100% identity) (57). The close relative of *Massilia brevitalea* was the most abundant species, comprising 20% of the bacterial population at a depth of 10.8 m, whereas *Psychrobacter glacincola* LMG21273 dominated the sediment, constituting 41% of the bacterial population at 13.8 m. A close relative of *Massilia aurea* AP13 (100% identity), a Gram-negative betaproteobacterium capable of starch degradation (58), was found at all depths except 7.7 m. The highest concentration of sediment arsenic in the present study was at 16.9 m and 26 m, where a close relative of the *Massilia aurea* AP13 formed 28% and 24%, respectively, of the total bacterial population.

The 16S rRNA gene sequences were very closely aligned with *Arthrobacter humicola* (100% identity), a Gram-positive actinobacterium (59), ranging from a high abundance of 18% of the total microbial community at a depth of 10.8 m to a low abundance of 1.8% at 26 m. *Comamonas aquatica* (99% identity), a Gram-negative betaproteobacterium (60), was found only in sediment with a relatively high arsenic concentration at 16.9 m, constituting 12% of the bacterial population in this case. While certain species of *Arthrobacter* are known for nitrate reduction, *Comamonas* species are known for oxidative carbohydrate metabolism with oxygen as an electron acceptor. The bacterial representatives in each sample, their relative abundance, and identity for Bangladesh site F are consistent with a broad range of processes, including both aerobic and anaerobic metabolism (see the supplemental material at https://doi.org/10.17632/3xrtpgdvfj.1).

The BLAST search for operational taxonomic units (OTUs) representing less than 1% of the microbial community indicated the presence of a diverse range of bacteria potentially colonizing the sediments using many contrasting forms of metabolic activity. Of the various representatives of microbes, the key bacteria potentially involved in the metabolism of iron, arsenic, and nitrate are discussed below.

### (ii) Iron-reducing bacteria.

Under anoxic conditions, the reduction of Fe(III) has been implicated in the mobilization of arsenic in aquifer sediments, through the reductive dissolution of As-bearing Fe(III) oxides and/or supporting the growth of organisms able to respire Fe(III) and also As(V) to the more mobile As(III) (11, 13). At least four relatives to known Fe(III)-reducing bacteria were found at all sediment depths in site F in low abundance (<1%). *Rhodobacter capsulatus* (98% identity), reported to be a dissimilatory Fe(III)-reducing bacteria (61), was detected at depths of 7.7, 13.8, and 16.9 m with a composition of below 0.2% of the total population for each depth. *Clostridium butyricum* EG3 (98% identity), another organism previously associated with dissimilatory Fe(III) reduction (62), was also found at depths of 19.9 m (0.01%), 22.9 m (0.04%), and 26 m (0.02%). Other bacteria well known to respire Fe(III) were present in low abundance across various depths, including close relatives to *Geobacter* spp., which are also known to respire As(V) (30). Given the potential link between the *Geobacter* species and the respiration of both Fe(III) and As(V) (61, 63), PCR amplification with the 584F/840R primer set specific for the 16S rRNA genes of the members of the *Geobacteraceae* family was also used in this study. PCR products were obtained in 6 samples out of the 8 tested, confirming the widespread presence of these Fe(III)-reducing bacteria in the iron-rich sediments (see Fig. S2B in the supplemental material).

### (iii) Arsenic-metabolizing bacteria.

At least 8 bacterial species closely related to those known to metabolize arsenic were identified at an abundance below 1% across various depths in the aquifer sediments at site F and included respiratory arsenate reducers (carrying the *arr* gene) implicated in mobilizing arsenic (30), detoxifying arsenate reducers (carrying *ars* genes), and close relatives to the aerobic arsenite oxidizers carrying the *aio* genes. For example, a close relative to *Pseudomonas putida* strain WB (100% identity), a dissimilatory arsenic-respiring bacterium (carrying both *arrA* and *arsC* genes [64]), was found at depths of 7.7, 16.9, and 26 m comprising less than 0.1% of the population. An organism most closely related to *Geobacter lovleyi* (93% identity), a dissimilatory Fe(III)-reducing bacterium that is also implicated in respiring As(V) (65), was found at depths of 7.7, 10.8, and 16.9 m at a relative abundance of below 0.1%. A close relative to the arsenate-respiring bacterium *Bacillus selenatarsenatis* (95% identity) (66, 67) was also found at depths of 7.7, 10.8, and 16.9 m, forming a community composition below 0.1%. A close relative to *Hydrogenophaga* sp. strain CL3 (98% identity) (68), a bacterium containing the *aroAB* genes responsible for oxidizing As(III), was found at depths of 7.7, 10.8, and 16.9 m comprising less than 0.1% of the total bacterial population, while a close relative of *Sinorhizobium* sp. (99% identity), another As(III)-oxidizing bacterium with the *aioA* gene, was also found (69) at the same depths below 0.1%.

### (iv) Nitrate- and nitrite-metabolizing bacteria.

Nitrate reduction is a widespread respiratory process that can also influence the fate of arsenic under anaerobic conditions, via the nitrite-driven oxidation of Fe(II) and As(III) (46, 47). As previously mentioned, close relatives to known nitrate-reducing bacteria, including *Massilia brevitalea*, *Psychrobacter glacincola*, *Arthrobacter defluvi*, and *Pseudomonas antarctica*, were found in high abundance (2% to 40%) across all depths, indicating the widespread potential for this form of metabolism in the sediments at site F. In addition, nitrifying bacteria were detected, such as a close relative to *Nitrospora* sp. (100% identity; found at depths of 7.7, 13.8, 16.9, and 22.9 m and comprising below 0.2% of the community structure), helping close the nitrogen redox cycle (70). Two OTUs very closely related to the *Bradyrhizobium* (100%), a genus of nitrifying bacteria that are known to convert nitrogen into ammonium, were found in low abundance at the same depths (71).

### (v) Sulfate-reducing bacteria.

Microbial sulfate reduction can be an important process in mitigating arsenic mobilization via the precipitation of poorly soluble arsenic-bearing sulfide phases. Sulfate-reducing bacteria were detected at various depths at site F, including examples affiliated with the genera *Desulfosporosinus* (72) at 10.8 m (0.05% relative abundance) and 16.9 m (0.03%), *Desulfobacca* (73) at 13.8 and 16.9 m (0.02% each), and the family *Syntrophobacteraceae* (73) at depths of 7.7 (0.1%) and 10.8 m (1.25%), respectively.

### (vi) PCR confirmation of functional genes.

To help further identify the sites of colonization of the As(V)-reducing bacteria, several primer sets were also used to target the alpha subunit of the *arrA* gene required for the dissimilatory arsenate reduction. Of the 5 different sets of primers used, only nested PCR using primers AS1F, AS1R, and AS2F amplified the gene successfully from sediments and positive controls—in this case using a seminested PCR technique. The *arrA* gene was detected in the samples collected at depths of 7.7, 10.8, 16.9, and 26 m. Figure S1A in the supplemental material accounts for the sample depths, where the *arrA* genes were amplified.

10.1128/mBio.01326-17.1 FIG S1  PCR confirmation of functional genes. The positive control (+ve) for the *arrA* gene is *Shewanella* sp. strain ANA-3, whereas the positive control for *Geobacteraceae* specific 16S rRNA gene and *dsr* gene is *Geobacter sulfurreducens*. The sizes of the amplified products are 625 and 1,900 bp, respectively. Download FIG S1, TIF file, 1.3 MB. Copyright © 2017 Gnanaprakasam et al. 2017 Gnanaprakasam et al. This content is distributed under the terms of the Creative Commons Attribution 4.0 International license.

The *dsr* gene found in dissimilatory sulfate-reducing bacteria, which can precipitate arsenic as sulfide phases (47, 74), was also amplified successfully using the primer set Dsr1F/Dsr4R from sediment depths of 7.7 and 10.8 m (Fig. S1C).

### Site B. (i) Sediment chemistry.

Site B is capped with a fine-grained silt and clay layer for the first 6 m and has a slower recharge rate of 0.08 m year^−1^. The arsenic concentration in 8 sediment samples varied from 1 mg kg^−1^ at 7.6 m to 6 mg kg^−1^ at 16.8 m. From 11 to 14 m, the arsenic concentration remained stable at 3 mg kg^−1^. The Fe concentration in the sediment was highest at 7.6 m, reaching 30.2 g kg^−1^, while the lowest concentration of 11.2 g kg^−1^ was recorded at a depth of 11 m. The concentration of Mn ranged from 178 mg kg^−1^ at 11 m to 612 mg kg^−1^ at 7.6 m, correlating well with the concentrations of Fe measured at the same depths (Fig. 2A).

Solid-phase arsenic speciation was determined by X-ray absorption near edge structure (XANES) at 13 depths on cuttings and core samples from 1.5 to 18.2 m below the ground surface (bgs). From 1.5 to 6 m bgs, the solid-phase As was dominated by As(V). The percentage of As(V) decreased below 6 m bgs, and As_2_S_3_ reached a maximum in the core samples at 7.6 m bgs. Below 7.6 m bgs, the sediment was dominated by As(III), except at 12.2 and 15.5 m bgs, where As(V) was present (Fig. 2B). The mineralogy of authigenic Fe minerals showed some broad similarities to those from site F, with ferrihydrite and goethite levels declining from 3 to 7.6 m bgs, indicating Fe(III) reduction, with siderite then detected at 10.97 and 13.7 m bgs. The Fe(II, III) silicates, modeled with biotite and hornblende, also remained stable throughout the depth profile (Fig. 2D).

### (ii) Aqueous chemistry.

The aqueous chemistry was analyzed in 3 water samples from the wells at site B. At a well depth of 7.3 m (BW0-3), the soluble arsenic concentration was 24 µg liter^−1^, rising to 290 and 460 µg liter^−1^ at depths of 10.8 m (BW10-8) and 14.3 m (BW14-3), respectively, far in excess of the WHO guideline of 10 µg liter^−1^. The Fe concentrations in water samples from the wells ranged from 15 mg kg^−1^ (270 µM) at a well depth of 7.3 m to 20 mg kg^−1^ (350 µM) at a depth of 14.3 m, which correlated well with the Fe concentrations in the corresponding sediment samples and was proportional to the arsenic concentrations detected in the water (Fig. 2E).

### Microbial ecology. (i) 16S rRNA community analysis.

The total number of processed 16S rRNA gene sequences obtained from site B by pyrosequencing was 55,759. The highest number of reads was obtained at a depth of 11 m (9,794 reads), and the lowest number was from a depth of 7.6 m (6,097 reads). The highest diversity of species was observed at a depth of 7.6 m with 1,215 distinct OTUs, whereas the lowest number of distinct OTUs was observed at a depth of 7.6 m (cut) with 74 species (Fig. 2F).

At site B, similarly to site F, *Acinetobacter* species were also relatively common, with a close relative to *Acinetobacter* sp. strain A32 (99.5% identity), another arsenic-resistant gammaproteobacterium (75), found in all depths except at 7.6 m, with an abundance ranging from 3% at a depth of 13.7 m to 17% at 10.7 m. Uniquely at 16.8 m, both *Acinetobacter* sp. strain A32 (99.0% identity) and *Brevundimonas* sp. strain A21-66 (99.3% identity), another alphaproteobacterium reported to be found in arsenic-contaminated soil and capable of reducing nitrate (76), were recorded as correlating with the highest concentration of arsenic that we detected in the sediments from this study (6 mg kg^−1^). A close relative of *Planococcus* sp. strain A08 (99.7% identity), an arsenic-resistant *Bacillus* reported to grow in concentrations of up to 640 mM arsenate and up to 14 mM arsenite, was detected at 10.7 m at a relative abundance of 1.4% (75).

*Massilia* species, known for nitrate reduction, were detected in relatively high abundance at four depths of 7.6, 10.7, 14, and 16.8 m. A close relative of *Massilia* sp. strain 51Ha (98.0% identity) (77) was the most abundant bacterium at a depth of 7.6 m, with an abundance of 9.4%. *Massilia brevitalea* (97.4% identity) was one of the most abundant species at 7.6 and 14 m, with abundances of 2.7 and 43%, respectively. These betaproteobacteria are known to denitrify, in common with the close relative to the gammaproteobacterium *Psychrobacter glacincola* (98.7% identity), which was found in abundance at depths of 7.6, 10.7, and 11 m with compositions of 3.0, 25 and 39%, respectively. A close relative of *Herbaspirillum* sp. strain P-64 (98.0% identity), a betaproteobacterium (78), was also found in all depths except 7.6 m, with abundances of 1.0% at 7.6 m, 20% at 10.7 m, 4% at 11 m, 33% at 13.7 m, 19% at 14 m, and 63% at 16.8 m. Some species of *Herbaspirillum* are known to carry the *arsC* gene coding for detoxification arsenate reductase (105) and also carry genes encoding respiratory and assimilatory nitrate reductases (NAR and NAS, respectively) (79). Also found in these sediment samples were common soil bacteria, including the *Duganella*, *Chryseobacterium*, *Exiguobacterium*, *Arthrobacter*, and *Trichococcus* species. The different representatives of the bacterial communities and their relative abundance found in site B are given in the supplemental material at https://doi.org/10.17632/3xrtpgdvfj.1.

A BLAST search for OTUs detected at below 1% of the sequences revealed the presence of diverse bacteria related to different metabolic activities in the sediment at site B, again including those potentially involved in the metabolism of iron, arsenic, and nitrate.

### (ii) Iron-metabolizing bacteria.

Close relatives to known Fe(III)-reducing bacteria were also found at all sediment depths at site B. In common with site F, *Rhodobacter* species were found at all depths except 10.7 m. The abundance varied from 0.02% at 7.6 m to 0.07% at 16.8 m. A close relative of the Fe(III) reducer *Pelobacter carbinolicus* (98% identity) (80) was also detected at a low relative abundance of below 1% at depths of 7.6 and 14 m. A close relative to the *Gallionella capsiferriformans* strain ES-2 (99% identity) (81), an Fe(II)-oxidizing bacterium that potentially closed the Fe redox cycle, was detected at an abundance of 0.03 to 0.04% at depths of 7.6, 10.7, and 16.8 m, respectively. The presence of *Geobacter*-related sequences was also noted, and *Geobacteraceae*-specific 16S rRNA gene primers showed the presence of these organisms in 5 of the 7 site B samples that were analyzed (Fig. S1B).

### (iii) Arsenic-metabolizing bacteria.

A range of potential arsenic-metabolizing bacteria were identified, including an organism most closely related to *Geobacter lovleyi* (92% identity), the dissimilatory As(V) [and Fe(III)]-respiring bacterium that was detected at 7.6 and 16.8 m with an abundance below 0.1% and noted in the key samples at site F. A close relative of *Pseudomonas putida* carrying *arrA* and *arsC* (99% identity) (82) was also detected in samples from the same depths of 7.6 and 16.8 m with an abundance below 1%. *Arthrobacter aurescens* (100% identity), a heterotrophic arsenate-reducing bacterium (83), was also found at all depths. *Stenotrophomonas* sp. strain MM7 (100% identity), an arsenite-oxidizing bacterium (84) that has the potential to detoxify As(V) via the Ars system, was also detected at depths of 14 and 16.8 m with an abundance below 0.1%. *Hydrogenophaga* sp. strain CL3 (100% identity), another “detoxifying” arsenite-oxidizing bacterium (68), was also detected at depths of 10.7, 13.7, and 16.8 m with a low abundance of below 1%.

### (iv) Nitrate- and nitrite-metabolizing bacteria.

In common with site F, there was evidence of the widespread colonization of site B sediments with nitrogen cycling bacteria. As previously noted, close relatives of *Psychrobacter glacincola* (97.8% identity) and *Massilia brevitalea* (97.4% identity), two nitrate-reducing bacteria, were found across various depths in high abundance of up to 50%, indicating an active metabolism related to nitrate reduction (55, 56). *Rhodobacter* spp. (99% identity), as previously mentioned, also implicated in Fe(III) reduction but well known to respire nitrate, were also commonly found at a low abundance of below 1% at depths of 7.6 and 13.7 m. A close relative to nitrite-oxidizing *Bradyrhizobium* sp. (99% identity) (71) was detected at below 1% of the community at 7.6, 10.7, 14, and 16.8 m. A close relative to *Nitrospira moscoviensis* (99% identity), another nitrite-oxidizing bacterium (85), was also detected at these depths at an abundance of below 1% of the bacterial community.

### (v) Sulfate-reducing bacteria.

Sulfate-reducing bacteria were also detected in low abundance at site B, typically below 1% of the total bacterial population and affiliated with the deltaproteobacteria and *Nitrospira*. Representatives of families implicated in sulfate reduction, including *Desulfarculaceae* and *Desulfobulbaceae*, were found at depths of 7.6 and 16.8 m (0.03% and 0.2%, respectively), while members of the *Desulfovibrionaceae* were found at 7.6, 13.7, and 16.8 m (0.03, 0.06, and 0.03%) and the *Desulfuromonadaceae* at 7.6 m (0.1%) (86). Sulfate-reducing bacteria from the *Syntrophobacteraceae* (73) were found in very low abundances of 1.4, 0.1, and 0.2%, respectively, at 7.6, 13.7, and 16.8 m.

### (vi) PCR confirmation of functional genes.

The primer sets AS1F/AS2F-AS1R, targeting the *arrA* gene required for dissimilatory arsenic-respiring bacteria, amplified the target gene successfully in sediment samples collected at depths of 7.6 and 16.8 m, where the arsenic concentrations in the sediment were 5 and 6 mg/kg, respectively. At all other depths, the arsenic-respiring genes were not amplified. Figure S1A shows the sample depths at which the *arrA* genes were amplified. Finally, primer set Dsr1F/Dsr4R, targeting the *dsr* gene conserved in dissimilatory sulfate-reducing bacteria, amplified the target gene at sediment depths of 7.6, 13.7, and 16.8 m (Fig. S1C).

### (vii) Correlation between the microbial and mineral interphase.

**FIG 3:**
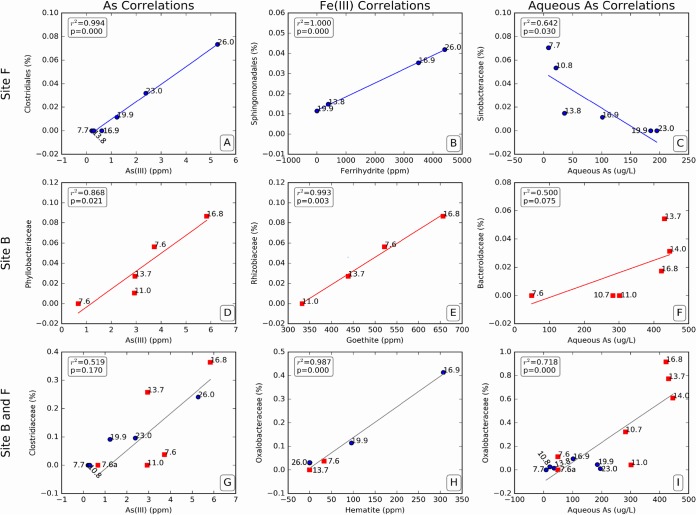
Top Spearman rank correlation (*P* = 0.01) plots relating microbial communities to mineral species. (A, D, and G) As(III) correlation with the microbial communities at site B, site F, and at combined sites. (B, E, and H) Fe(III) correlation with the microbial communities at site B, site F, and at combined sites. (C, F, and I) Aqueous As correlation with the microbial communities.

Given the complexity of the microbial communities detected by next-generation sequencing, and the comprehensive geochemical and mineralogical data sets collected from our two field sites, Spearman rank correlations were used in order to identify key phylogenetic groups that were associated with the changes in mineralogy and arsenic solubility at our field site. A synopsis of these results (Fig. 3) shows the top correlations at sites F and B between relative abundance and As(III), Fe mineralogy, and the concentrations of aqueous arsenic. A more detailed analysis is included in Table S3 and the correlation figures from Fig. S2 to Fig. S7 in the supplemental material.

10.1128/mBio.01326-17.10 TABLE S3  Correlations between mineralogy and the molecular data. Download TABLE S3, DOCX file, 0.1 MB. Copyright © 2017 Gnanaprakasam et al. 2017 Gnanaprakasam et al. This content is distributed under the terms of the Creative Commons Attribution 4.0 International license.

10.1128/mBio.01326-17.2 FIG S2  Top 9 correlation of bacteria with Fe and As at site F. Download FIG S2, TIF file, 2.3 MB. Copyright © 2017 Gnanaprakasam et al. 2017 Gnanaprakasam et al. This content is distributed under the terms of the Creative Commons Attribution 4.0 International license.

10.1128/mBio.01326-17.3 FIG S3  Top 9 correlation of bacteria with arsenic at site F. Download FIG S3, TIF file, 1.2 MB. Copyright © 2017 Gnanaprakasam et al. 2017 Gnanaprakasam et al. This content is distributed under the terms of the Creative Commons Attribution 4.0 International license.

10.1128/mBio.01326-17.4 FIG S4  Top 9 correlation of bacteria with Fe and As at site B. Download FIG S4, TIF file, 1.4 MB. Copyright © 2017 Gnanaprakasam et al. 2017 Gnanaprakasam et al. This content is distributed under the terms of the Creative Commons Attribution 4.0 International license.

10.1128/mBio.01326-17.5 FIG S5  Top 9 correlation of bacteria with arsenic at site B. Download FIG S5, TIF file, 1.1 MB. Copyright © 2017 Gnanaprakasam et al. 2017 Gnanaprakasam et al. This content is distributed under the terms of the Creative Commons Attribution 4.0 International license.

10.1128/mBio.01326-17.6 FIG S6  Top 9 correlation of bacteria with Fe and As at sites F and B. Download FIG S6, TIF file, 1.3 MB. Copyright © 2017 Gnanaprakasam et al. 2017 Gnanaprakasam et al. This content is distributed under the terms of the Creative Commons Attribution 4.0 International license.

10.1128/mBio.01326-17.7 FIG S7  Top 9 correlation of bacteria with arsenic at sites F and B. Download FIG S7, TIF file, 1.2 MB. Copyright © 2017 Gnanaprakasam et al. 2017 Gnanaprakasam et al. This content is distributed under the terms of the Creative Commons Attribution 4.0 International license.

At site F, 133 out of 502 (26%) species were correlated with a *P* value of <0.01 to a mineralogical or redox-sensitive parameter, for a total of 228 correlations, with 8 species with 2 correlations and 25 species with 3 correlations. Of these, 85% of the correlations were to a solid-phase parameter, 5% to a liquid-phase parameter, and 9% to both. In the solid phase, most correlations were to As(III), As(V), and sediment reflectance, with fewer correlations to Fe minerals (Table S3A; Fig. S2).

At site B, 129 out of 561 (23%) of the species were correlated with a *P* value of <0.01 to a mineralogical or redox-sensitive parameter, for a total of 189 correlations with 55 species with 2 correlations and 14 species with 3 correlations. A total of 85% of the correlations were to a solid-phase parameter, 13% to a liquid-phase parameter, and 2% to both. In the solid phase, most correlations were to biotite, ferrihydrite, and goethite, with fewer correlations to the arsenic phases (Table S3A; Fig. S4).

When the sites were combined and only the species present at both sites were considered, 73 out of 274 (26%) of the species were correlated with a *P* value of <0.01 to a mineralogical or redox-sensitive parameter, for a total of 139 correlations with 33 species with 2 correlations and 3 species with 3 correlations. Forty-five percent of the correlations were to a solid-phase parameter, 18% to a liquid-phase parameter, and 37% to both. Combining both sites increased the percentage of aqueous phase correlations in comparison to the solid phase. The highest numbers of correlations were to aqueous Fe, As(V) (solid phase), and aqueous arsenic. Figure 3 presents the top 9 correlation plots that depict the microbial community correlations with key mineralogical and geochemical species. Of particular interest is the correlation of As(III) and the Gram-positive bacteria affiliated with the order *Clostridiales*, which is normally associated with the anaerobic degradation of organic matter but were not previously noted to be able to respire As(V). It may be that their role in arsenic release is due to their ability to degrade complex organic matter, which drives microbial As(V) reduction by more specialized metal-reducing bacteria, but a potentially more direct involvement warrants further investigation. Focusing on data from site F (Fig. S3), similar hypotheses could be tested for close affiliates of the *Bacteroides* species, correlated with high arsenic concentrations. Thus, these correlations might suggest previously unidentified physiological traits, and this concept could be extended to the identification of potential Fe(III)-reducing bacteria affiliated with the genera *Sphingomonas* and *Rhizobium*, which were correlated with ferrihydrite and goethite, respectively.

## DISCUSSION

Two shallow, high-arsenic aquifer sites were analyzed in order to determine sediment-phase arsenic speciation and the corresponding microbial assemblages with depth across an arsenic gradient. The depth gradients do not trace one groundwater flow path from the surface to depth but instead are used as a proxy to represent the evolution of groundwater. The two sites were chosen for the contrasting hydrology and geology. Site F has sand to the surface with rapid recharge, irrigation pumping nearby, and a relatively deep arsenic maximum at 18.4 m, while site B is capped by a thick clay, has slower recharge rates, and has a shallower and higher arsenic maximum. At both sites, the solid-phase arsenic was dominated by As(V) near the surface, where the groundwater is low in arsenic. At depths where the groundwater was elevated in arsenic, the sediment was predominantly As(III). This consistent trend across these sites suggests that this redox reaction plays a role in controlling the observed groundwater arsenic levels.

These results are broadly consistent with previous studies (38–40, 87) which suggest that the number of sorption sites for arsenic will be diminished, where Fe(III) minerals are respired at depth and converted to sorbed Fe(II) and ferrous minerals, and the binding of any arsenic present will be skewed toward the accumulation of any residual As(V), rather than As(III), which would be expected to accumulate in the aqueous phase. The conversion of As(V) to As(III) at depth is clear from our XAS data at both sites, and the reduction of Fe(III) minerals with depth is particularly striking for site B. At both sites, the solid-phase arsenic was dominated by As(V) near the surface, where the groundwater is low in arsenic. At depths where the groundwater was elevated in arsenic, the sediment was predominantly As(III). This consistent trend across these sites suggests this redox reaction plays a role in controlling the observed groundwater arsenic levels.

The microbial ecology of the two contrasting sediments was studied by means of high-throughput 16S rRNA gene pyrosequencing and functional gene probing via PCR. These approaches confirmed a rich diversity of organisms potentially able to catalyze a wide range of biogeochemical reactions that could impact both arsenic speciation and solubility. 16S rRNA gene profiling revealed that known representatives of both dissimilatory Fe(III)- and As(V)-reducing bacteria were widespread throughout the sediment profiles at both sites, including those affiliated with the family *Geobacteraceae*, implicated in microcosm studies in mobilizing As from other sedimentary settings (11, 88, 89). This widespread distribution of organisms that could potentially respire As(V), producing the more mobile As(III) detected at depth, was further mirrored by the prevalence of the *arrA* gene detected throughout the profiles at both sites. Interestingly, there was also evidence for the presence of bacteria across the profiles, which given the right geochemical conditions could attenuate arsenic mobilization, including sulfate-reducing bacteria that are able to precipitate arsenic as As_2_S_3_ (47, 90), although their detection did not always coincide with the presence of arsenic sulfides in the sediments. This was especially true at site F, although at site B (which had a lower recharge rate), the *dsr* gene was successfully amplified at two depths where As_2_S_3_ was detected by XANES. Even at these potential “hot spots,” they did not dominate the arsenic speciation. Nitrate reduction can also be invoked in the control of arsenic speciation, being coupled to the oxidation of Fe(II) and As(III), resulting in the precipitation of As(V)-bearing Fe(III) oxides (47). Once more, nitrate reducers were present throughout the samples analyzed, although it was clear that with depth As(III) dominated in the solid phase and the levels of soluble arsenic increased.

Overall, the extant organisms and the biogeochemical processes that they catalyze seem to be comparable for both study sites, despite their contrasting hydrologies. As(V) and Fe(III) are reduced at depth, most likely leading to their release to groundwater and also in As(III) dominating in the solid phase. Competing biogeochemical processes, such as denitrification and sulfate reduction (which can capture arsenic), and natural attenuation via sorption, are clearly not capable of preventing the accumulation of concentrations of arsenic well above the WHO guideline. Although this study cannot unequivocally identify the causative organisms or identify the mechanisms of arsenic release, *Geobacter* species known to reduce both Fe(III) and As(V) and implicated in arsenic mobilization in previous studies were detected throughout and could play a critical role in arsenic release. In addition, approximately 25% of the species were correlated to a solid-phase arsenic species or an Fe mineral, indicating that a significant fraction of the microbial community is associated with these critical phases. The Spearman rank correlations identified new phylogenetic groups that could be linked directly or indirectly with As(V) reduction and mobilization, and identification of their potential role in such processes warrants further investigation. However, given the complex microbial communities identified and the relatively low abundance of known metal-reducing bacteria, the organisms causing these problems are likely to constitute a relatively minor component of the microbial communities. The mobilization of arsenic in carbon-stimulated microcosms and pure culture lab experiments (12, 14, 34) can be rapid (on the order of weeks). It is likely that under *in situ* conditions, these processes take longer to deliver the arsenic into the aqueous phase, consistent with higher-arsenic waters that are several decades old (12). This would also be consistent with lower organic loadings of bioavailable organic material expected at depth and the relatively low abundance of metal-reducing bacteria in the microbial communities detected. The precise nature of the organic matter fueling metal reduction at depth clearly requires further investigation, as does the potential role of other potential electron donors, such as ammonium, in such processes (91).

Mechanisms at play at the two sites could include the release of As(V) during the reductive dissolution of As(V)-bearing Fe(III) oxides, followed by the reduction of the liberated As(V) by the periplasmic Arr system and then the resorption of As(III) (with some remaining in the solution). Other mechanisms that could play a role include the direct solid-phase reduction of As(V), noted in pure culture studies (92), and the mobilization of some of the resultant As(III). Although this is not consistent with the direct role of a periplasmic enzyme system (mediated by Arr), the extracellular reduction of metals via humics, secreted flavins, and nanowires protruding from the cell surface is becoming an excepted paradigm (93), and electron transfer by means of mineral assemblages could also play a role. It should also be noted that the solid-phase biomineral capture of both As(III) and As(V) is also well known from laboratory studies—e.g., to Fe(II) minerals (20, 36, 94)—and could be invoked *in situ*. The microbial processes controlling arsenic fate in complex aquifer systems, such as those operating at these Bangladeshi field sites, clearly warrant further study and will benefit from the application of new techniques that will allow the identification of active microbes mediating arsenic release *in situ* and the genes involved. This is an accepted limitation of the targeted DNA-focused approaches that we have used in this study, which do not exclusively target the active microbial community *in situ*. This could be addressed by encompassing transcriptomic profiling techniques, which is a goal for our future studies on these systems. Metagenomic analyses could also help identify the key metabolic processes that drive the system, including autotrophy and the heterotrophic metabolism of the electron donors, and the role of coupled and competing processes, such as Fe(III) reduction and methanogenesis, respectively.

## MATERIALS AND METHODS

### Study site.

The study sites are located in Araihazar Upazila, Bangladesh, approximately 25 km east of the capital, Dhaka. The sites are located in Lashkardi village (site F) (23.774°N, 90.606°E) and Baylarkandi village (site B), (23.780°N, 90.640°E). Tube wells were previously installed at these arsenic-impacted sites in 2001 in order to monitor the temporal variability (6, 9). Site F is a sandy site with sand extending to the surface. The recharge rate is 0.5 m year^−1^, and the maximum arsenic concentration of 200 μg liter^−1^ is reached at a depth of 26 m (95). Site F drilling was performed 5 m from the tube wells in a hollow that was 1.6 m lower than the tube wells. Site B is capped by approximately 6 m of the fine-grained silt and clay that overlies the sandy aquifer. The recharge rate is 0.08 m year^−1^, and the maximum arsenic concentration of 460 μg liter^−1^ is reached at a depth of 13.7 m (95). Assuming a porosity of 0.25 at each site, the vertical groundwater velocities are 2.0 m year^−1^ at site F and 0.32 m year^−1^ at site B. While the water samples were collected from the existing tube wells installed in 2001, the sediment samples were acquired from a fresh coring at the same sites adjacent to the existing wells.

### Sample collection.

Sediment cuttings and cores were collected using the “hand-flapper” method, a manual drilling method often used to install wells (8, 96). Cuttings were collected from the sediment as it exited the drill pipe. Intact sediment cores were collected at approximately 1.52-m (5-ft) intervals to 18.3 m (60 ft) at site B and 26.0 m (80 ft) at site F, by lowering a 1- or 2-in. inside-diameter (i.d.) gravity corer inside the drill pipe and into the sediment that had not yet been drilled. Upon coring, the sediment samples were split into aliquots for different types of analyses. The samples for the XAS analysis were saturated with glycerol (approximately 1:1 [vol/vol]) as a preservative and stored at −20°C prior to analysis. Glycerol is added to prevent oxidation by slowing the oxygen diffusion and because it fixes bacteria in order to prevent microbiological alteration. In this case, spectra were collected using a thin (a few millimeters) film of wet sediments without further treatment. The sample collection and synchrotron access were coordinated in order to minimize the lag time (about 2 weeks) between sampling and analysis. The samples used for the X-ray fluorescence (XRF) were used immediately in the field (without treatment), and the samples for the other analyses were stored in the refrigerator prior to analysis. From each depth, approximately 5 g of sediment was incised aseptically in an anaerobic bag fluxed with N_2_ and stored anaerobically at −20°C for molecular investigation. The samples were hermetically sealed and transported to the respective laboratories for analysis. The diffuse spectral reflectance of fresh cuttings was also measured in the field as a proxy for the Fe(II)/Fe(II + III) ratio in the acid-leachable Fe fraction (8).

Groundwater samples were collected from 5 tube wells from site F and 3 tube wells from site B. A battery-driven submersible pump (Whale SuperPurger) was used in order to pump the water from the wells at a rate of about 2 liters/min. The samples for arsenic, other trace elements, and other cations were collected in 30-ml acid-cleaned high-density polyethylene (HDPE) bottles, as described previously (6, 9).

### Sediment chemistry. (i) XRF.

The elemental composition of the sediment was determined by means of X-ray fluorescence (XRF) spectroscopy using an InnovX Delta Premium hand-held XRF analyzer using triplicate measurements and a collection time of approximately 75 s. This device calculates environmental concentrations based on total fluorescence at three energy levels in order to efficiently discriminate between elements, such as lead and arsenic, with similar emission lines. The levels of analytic precision based on statistical counting errors are approximately ±1 to 2 mg kg^−1^ and ±300 mg kg^−1^ for arsenic and Fe, respectively. Under the same conditions, the levels of analytic precision of the repeated measurements of uniform standard reference materials collected independently over days to weeks are within 5% of each other and are within 6% of the certified values. Since the XRF only samples a small quantity of the sample, the measured concentrations can vary more significantly when repeated on heterogeneous natural materials, and analytic accuracy under the experimental conditions is thus somewhat lower: about 10 to 20% for any single measurement.

### (ii) EXAFS spectra for iron and arsenic.

Approximately 0.1 g of sediment from each depth was analyzed for Fe, and an arsenic extended X-ray absorption fine structure (EXAFS) analysis was performed at the Stanford Synchrotron Radiation Laboratory (SSRL) on beamlines 4-1 and 4-3, equipped with a 13- and 32-element Ge detector, respectively. The beamlines were configured with an Si(220) monochromator with a phi angle of 90°. Soller slits were installed in order to minimize the effects of the scattered primary radiation. The beam was detuned at least 50% to reject higher-order harmonic frequencies and prevent detector saturation. Scans were calibrated to the Fe K-edge of As(V) (11,874.0 eV for sodium arsenate) and Fe metal (7,112 eV) using a sample placed between the second and third ionization chambers. A subset of each sample was sealed in Kapton tape and mounted between the first and second ionization chambers, and sample spectra were obtained in fluorescence mode in combination with a 6-μx Ge filter (for the analysis of arsenic) or Mn filter (for the analysis of Fe).

Spectra were processed in SIXpack (106) unless mentioned otherwise. Arsenic K-edge XANES spectra were normalized and fit by linear combination fitting (LCF) using orpiment (As_2_S_3_), arsenite [as adsorbed arsenite; As(III)], and arsenate [as adsorbed arsenate; As(V)] (97). Other arsenic oxidation states were considered but were not required for any fits. Adsorbed references were created by adsorbing either sodium arsenate or sodium arsenate solutions on 1-g/liter ferrihydrite solutions to a solid concentration of 1,000 mg arsenic/kg Fe.

Fe mineralogy was determined using the LCF fitting of the EXAFS spectra with those of the reference spectra of the commonly occurring sediment minerals (hematite, goethite, ferrihydrite, magnetite, mackinawite, siderite, biotite, and hornblende) (97). Averaged Fe K-edge EXAFS spectra were normalized using linear pre-edge and quadratic post-edge functions. Normalized spectra were converted to *k*^3^-weighted chi functions with a threshold energy (E0) of 7,124 eV. The reference spectra to be included in linear combination fitting were selected based on the minerals known to be present or commonly present in sediments and also included minerals often found by other methods (for example, by diffraction or magnetic susceptibility). The principal-component analysis (PCA) was also applied on sample *k*^3^-weighted spectra in order to select reference spectra. Target transforms were used to compare signiﬁcant components against a spectral library of Fe mineral references in order to ensure that only references that were deemed statistically viable were included. Only target transforms having SPOIL values of <6 were considered potential references (107, 108). In the end, all spectra were fit with a single set of reference spectra, although individual samples likely contain minerals not represented in this reference set. This approach was implemented because it quantifies Fe mineralogy in a manner that is internally consistent and representative of the oxidation state, but it means that the fractional mineral concentrations of some minerals are combined and represented with a smaller set of similar reference spectra that are not easily differentiated. For example, the Fe(II) and Fe(III) silicates all have sufficiently similar spectra, which makes it hard to resolve them, and consequently fits involving several of these minerals concurrently are rendered unreliable. Thus, we limit fits to representative and common Fe(II) silicates such as biotite and Fe(II, III) silicates such as hornblende. As a result, care must be used in interpreting the mineralogical data as indicative of the relative concentration of specific minerals, in particular for Fe silicates. A least-squares LCF was implemented on the *k*^3^-weighted EXAFS spectra over a k-range of 2 to ~13 Å^−1^ in order to determine the relative fraction of each of the 8 reference compounds that contribute to the sample spectra. Uncertainties reported by SIXpack include the Monte Carlo-based error propagation from fitting, spectral noise in the sample and reference spectra, and similarities between the reference spectra (106). The fractions and uncertainties were reported as percentage of total Fe, if needed converted to concentrations by multiplying bulk Fe concentrations, and expressed in milligrams of Fe per kilogram of sediment.

### (iii) Aqueous chemistry.

High-resolution inductively coupled plasma mass spectrometry (ICP-MS) was used to analyze soluble arsenic, Fe, and other elements in well water (9, 98). The accuracy and the detection limits (<0.1 μg/liter) were verified against reference standards NIST1640A and NIST1643.

### Microbial ecology. (i) 16S rRNA-based bacterial community analysis.

The culture-independent community analysis was conducted using 16S rRNA gene sequencing protocols. The total DNA was isolated from the 1-g samples of sediment using the PowerSoil DNA isolation kit (Mo Bio Laboratories, Inc., Carlsbad, CA), following the manufacturer’s protocol. The quantity of DNA in the extracts was measured at 260 nm using a Nanodrop ND-1000 spectrophotometer (Thermo Scientific). Pyrosequencing used Roche’s FastStart high-fidelity PCR system with the forward primer 27F (99) and reverse primer 338R (100), which target the V1-V2 hypervariable region of the 16S rRNA gene in bacteria. Sequencing was performed using a Roche 454 Life Sciences GS Junior system at the sequencing facility at the University of Manchester. The 454 pyrosequencing reads obtained in FASTA format were analyzed using QIIME version 1.8.0 using the parameters of 97% sequence similarity in OTU picking and the 80% confidence threshold in taxonomic classification.

### (ii) Functional gene analyses.

In addition to the microbial community analysis using 16S rRNA gene primers, arsenate-respiring bacteria were targeted with a combination of functional gene primers that target the *arrA* gene, including AS1F/AS1R-AS2R (101), HA ArrA-D1F/HA Arra-G2R (102), ArrAUF1/ArrAUR3 (103), ArrAfwd/ArrArev, and CHArrAfwd/CHArrArev (28). The primer set Dsr1F/Dsr4R was also used to amplify the 1.9-kb functional gene (*dsr*) coding for the dissimilatory sulfite reductase (DSR) conserved among all known sulfate-reducing prokaryotes (74). The distribution of iron reducers was estimated using a PCR-based amplification using the 584F/840R primer set specific for the 16S rRNA genes of Fe(III)-reducing bacteria of the family *Geobacteraceae* (104). The primer details and the PCR conditions for the PCR for dissimilatory arsenate-reducing bacteria (*arrA* gene), the PCR for dissimilatory sulfate-reducing bacteria (*dsr gene*), and the PCR for the *Geobacteraceae* 16S rRNA gene can be found in the supplemental material at https://doi.org/10.17632/3xrtpgdvfj.1.

### Statistical analysis.

Correlations were determined between the independent parameters of geochemistry and the dependent parameters of the percentage of species relative abundance using the Spearman’s rank correlation with a critical significance level of 0.01 in order to minimize the effect of the outliers. The number of significant correlations was summed in order to understand controls on species abundance and to relate microbiological abundance to the mineralogical and solution composition.
