# Supplementary material for: Microbial Community Structure and Arsenic Biogeochemistry in Two Arsenic-Impacted Aquifers in Bangladesh
Source: mBio. 2017 Nov 28;8(6):e01326-17. doi: 10.1128/mBio.01326-17 (PMC5705915; doi:10.1128/mBio.01326-17)
Supplement: TABLE S1 [file mbo006173605st1.docx]

| **Table S-1. Chemical and molecular ecology analysis of sediments and water from the Site F aquifer*** | | | | | | | | | | | | | | | | | | | | |
| --- | --- | --- | --- | --- | --- | --- | --- | --- | --- | --- | --- | --- | --- | --- | --- | --- | --- | --- | --- | --- |
| **XANES-Arsenic** | | | | | | | | | | | | | | | | | | | | |
| **Sample ID** | | **FS03-1** | **FS04-7** | **FS06-2** | **FS07-7** | **FS07-7a** | **FS09-2** | | **FS10-8** | **FS12-3** | **FS13-8** | **FS13-8a** | **FS16-9** | **FS16-9a** | **FS18-4** | **FS19-9** | **FS21-4** | **FS22-9** | **FS24-5** | **FS26-0** |
| Depth (m) | | 3.1 | 4.7 | 6.2 | 7.7 | 7.7 cut | 9.2 | | 10.8 | 12.3 | 13.8 | 13.8 cut | 16.9 | 16.9 cut | 18.4 | 19.9 | 21.49 | 22.99 | 24.5 | 26.0 |
| As(III) (%) | | 3.7E-02 | 9.9E-02 | 8.2E-02 | 1.6E-01 | 2.1E-01 | 2.0E-01 | | 2.7E-01 | 4.2E-01 | 2.9E-01 | 2.9E-01 | 1.5E-01 | - | 5.5E-01 | 6.1E-01 | 9.0E-01 | 7.9E-01 | 1.1E+00 | 1.1E+00 |
| As(V) (%) | | 9.2E-01 | 9.0E-01 | 9.8E-01 | 8.8E-01 | 8.8E-01 | 8.3E-01 | | 7.5E-01 | 5.8E-01 | 7.1E-01 | 7.1E-01 | 8.3E-01 | - | 6.6E-01 | 1.1E-01 | 1.2E-01 | 1.8E-01 | 7.7E-08 | 1.3E-04 |
| As_2_S_3_ (%) | | 7.2E-09 | 7.6E-08 | 5.4E-08 | 3.2E-08 | 3.4E-03 | 5.5E-08 | | 2.7E-08 | 4.4E-08 | 4.7E-08 | 4.7E-08 | 6.1E-08 | - | 1.7E-08 | 3.8E-01 | 5.6E-08 | 1.8E-08 | 2.4E-08 | 4.3E-08 |
| **EXAFS-Fe minerals** | | | | | | | | | | | | | | | | | | | | |
| Siderite (%) | | 3.4E-09 | 2.3E-05 | 6.7E-09 | 1.2E-02 |  | 1.4E-08 | |  | 4.3E-08 | 1.6E-08 |  | 5.3E-08 |  |  | 3.9E-03 | 2.5E-09 |  | 8.0E-03 | 3.2E-04 |
| Goethite (%) | | 1.7E-01 | 1.0E-01 | 1.0E-01 | 7.0E-02 |  | 1.8E-01 | |  | 2.1E-01 | 2.7E-01 |  | 2.3E-01 |  |  | 9.5E-02 | 1.8E-01 |  | 4.5E-02 | 1.1E-01 |
| Hematite (%) | | 6.8E-09 | 5.5E-09 | 1.1E-08 | 1.9E-04 |  | 1.3E-02 | |  | 6.4E-03 | 1.3E-08 |  | 2.5E-02 |  |  | 7.9E-03 | 1.8E-08 |  | 1.2E-04 | 1.0E-08 |
| Magnetite (%) | | 1.6E-08 | 2.2E-07 | 1.5E-08 | 1.2E-08 |  | 5.8E-02 | |  | 3.6E-02 | 1.2E-09 |  | 2.0E-08 |  |  | 2.4E-02 | 1.7E-02 |  | 4.2E-07 | 1.1E-06 |
| Mackianite (%) | | 2.0E-09 | 2.8E-06 | 6.5E-09 | 1.9E-08 |  | 1.5E-06 | |  | 4.0E-04 | 3.4E-08 |  | 4.4E-02 |  |  | 1.5E-02 | 8.0E-04 |  | 2.4E-08 | 3.4E-03 |
| Biotite (%) | | 7.3E-02 | 1.6E-01 | 1.1E-01 | 2.2E-01 |  | 1.3E-01 | |  | 2.2E-01 | 4.3E-01 |  | 2.1E-01 |  |  | 3.4E-01 | 2.7E-01 |  | 3.5E-01 | 3.8E-01 |
| Hornblende (%) | | 3.1E-01 | 2.9E-01 | 3.0E-01 | 3.4E-01 |  | 3.1E-01 | |  | 4.8E-01 | 2.7E-01 |  | 2.1E-01 |  |  | 5.2E-01 | 3.0E-01 |  | 4.6E-01 | 3.8E-01 |
| Ferrihydrite (%) | | 4.5E-01 | 4.5E-01 | 4.9E-01 | 3.7E-01 |  | 3.0E-01 | |  | 4.3E-02 | 2.7E-02 |  | 2.9E-01 |  |  | 8.2E-08 | 2.4E-01 |  | 1.3E-01 | 1.2E-01 |
| **XRF - Sediments****** | | | | | | | | | | | | | | | | | | | | |
| As (mg/kg) | | 1 | 3 | 3 | - | 1 | 3 | | 1 | 1 | 1 | - | 4 | - | 6 | 2 | 1 | 3 | 3 | 5 |
| Fe (g/kg) | | 33.5 | 16.5 | 14.1 | - | 13.6 | 16.1 | | 9.7 | 14.9 | 14.5 | - | 12.1 | - | 9.7 | 12.1 | 9.3 | 11.8 | 11.8 | 35.3 |
| Mn (mg/kg) | | 581 | 346 | 257 |  | 297 | 312 | | 187 | 280 | 268 |  | 174 |  | 212 | 266 | 147 | 209 | 244 | 753 |
| **ICP-MS :Water** (depth: mid-screen is 1.5 m or ( + and - 0 .75 m) | | | | | | | | | | | | | | | | | | | | |
| **Sample ID** | |  |  | **FW5-8** |  |  |  | | **FW11-1** |  |  |  | **FW15-1** |  |  | **FW19-4** |  |  |  | **FW25-4** |
| As (ug/L) | |  |  | 0.544 |  |  |  | | 22.470 |  |  |  | 42.381 |  |  | 182.716 |  |  |  | 203.236 |
| Total Fe | uM |  |  | 0.151 |  |  |  | | 0.345 |  |  |  | 9.652 |  |  | 15.519 |  |  |  | 299.584 |
|  | mg/L |  |  | 0.008 |  |  |  | | 0.019 |  |  |  | 0.539 |  |  | 0.866 |  |  |  | 16.730 |
| Total S | uM |  |  | 65.197 |  |  |  | | 39.108 |  |  |  | 16.664 |  |  | 42.960 |  |  |  | 4.161 |
|  | mg/L |  |  | 2.091 |  |  |  | | 1.254 |  |  |  | 0.5343 |  |  | 1.377 |  |  |  | 0.133 |
| Total Mn | uM |  |  | 0.140 |  |  |  | | 20.892 |  |  |  | 22.416 |  |  | 42.052 |  |  |  | 31.103 |
|  | mg/L |  |  | 0.008 |  |  |  | | 1.147 |  |  |  | 1.231 |  |  | 2.310 |  |  |  | 1.708 |
| Tritium Age | |  |  |  |  |  |  | | 0.790 |  |  |  |  |  |  | 5.280 |  |  |  | 29.1 |
| **Molecular Analysis** | | | | | | | | | | | | | | | | | | | | |
| 16S rRNA gene | |  |  |  |  | √ |  | √ | |  | √ |  | √ | √ |  | √ |  | √ |  | √ |
| Geobacter -16S rRNA | |  |  |  |  | √ |  | √ | |  | √ |  | x | √ |  | √ |  | √ |  | x |
| As(V) reducers (arrA) | |  |  |  |  | √ |  | √ | |  | x |  | √ | x |  | x |  | x |  | √ |
| Sulphate reducers (*dsr*) | |  |  |  |  | √ |  | √ | |  | x |  | x | x |  | x |  | x |  | x |

* ‘*Blank cells’* in the table denote the absence of analysis for the respective sample, ‘√’ denotes the positive results based on PCR products of respective *16S rRNA* gene, Geobacter specific *16S rRNA* gene, and *arrA* gene for arsenate reducers and *dsr* gene for sulphate reducers; ‘x’ denotes the negative results or unamplified products .

**** The standard error for As, Fe and Mn were ≤ 1, ≤ 99 and ≤ 12, respectively.
