# Supplementary material for: Microbial Community Structure and Arsenic Biogeochemistry in Two Arsenic-Impacted Aquifers in Bangladesh
Source: mBio. 2017 Nov 28;8(6):e01326-17. doi: 10.1128/mBio.01326-17 (PMC5705915; doi:10.1128/mBio.01326-17)
Supplement: TABLE S2 [file mbo006173605st2.docx]

| **Table S-2. Chemical and molecular ecology analysis of sediments and water from the Site B aquifer*** | | | | | | | | | | | | | | | | | |
| --- | --- | --- | --- | --- | --- | --- | --- | --- | --- | --- | --- | --- | --- | --- | --- | --- | --- |
| **XANES : Arsenic** | | | | | | | | | | | | | | | | | |
| Sample ID | | **BS01-5** | **BS03-0** | **BS04-6** | **BS06-0** | **BS07-6** | **BS07-6a** | **BS10.0** | **BS10-7** | **BS10-7a** | **BS11-0** | **BS12-2** | **BS13-7** | **BS14-0** | **BS15-5** | **BS16-8** | **BS18-3** |
| **Depth (m)** | | **1.52** | **3.04** | **4.57** | **6.09** | **7.62** | **7.62** | **10.05** | **10.66** | **10.66** | **10.97** | **12.19** | **13.71** | **14.02** | **15.5** | **16.76** | **18.28** |
| As(III) (%) | | 7.0E-02 | 3.2E-02 | 1.5E-01 | 2.1E-01 | 7.4E-01 | 6.7E-01 | 7.5E-01 |  |  | 9.8E-01 | 4.9E-01 | 9.8E-01 |  | 3.0E-01 | 9.7E-01 | 1.0E+00 |
| As(V) (%) | | 9.7E-01 | 1.0E+00 | 8.0E-01 | 7.9E-01 | 4.8E-02 | 2.3E-01 | 2.3E-01 |  |  | 1.2E-01 | 5.3E-01 | 1.2E-02 |  | 7.0E-01 | 2.7E-08 | 1.6E-08 |
| As_2_S_3_ (%) | | 5.1E-08 | 5.5E-08 | 7.8E-02 | 1.8E-02 | 2.9E-01 | 1.0E-02 | 3.7E-08 |  |  | 8.8E-09 | 1.0E-07 | 1.0E-01 |  | 1.3E-08 | 1.4E-07 | 2.6E-08 |
| **EXAFS-Fe minerals** | | | | | | | | | | | | | | | | | |
| Siderite (%) | |  | 1.8E-08 | 1.6E-08 | 1.5E-08 | 1.3E-02 |  |  |  |  | 5.6E-02 |  | 4.7E-02 |  |  | 2.6E-08 | 1.0E-08 |
| Goethite (%) | |  | 1.3E-01 | 1.8E-01 | 1.3E-01 | 2.3E-02 |  |  |  |  | 2.9E-02 |  | 2.7E-02 |  |  | 1.9E-01 | 1.1E-01 |
| Hematite (%) | |  | 1.9E-02 | 1.1E-06 | 8.8E-05 | 1.4E-03 |  |  |  |  | 1.6E-08 |  | 1.2E-09 |  |  | 5.0E-09 | 9.0E-03 |
| Magnetite (%) | |  | 3.0E-05 | 1.9E-02 | 2.3E-04 | 9.8E-03 |  |  |  |  | 3.9E-02 |  | 9.8E-02 |  |  | 3.7E-02 | 5.8E-02 |
| Mackianite (%) | |  | 2.0E-09 | 1.0E-02 | 4.0E-09 | 4.2E-08 |  |  |  |  | 1.1E-03 |  | 8.1E-02 |  |  | 3.4E-02 | 2.3E-08 |
| Biotite (%) | |  | 7.6E-02 | 1.5E-01 | 3.8E-01 | 3.7E-01 |  |  |  |  | 2.8E-01 |  | 2.8E-01 |  |  | 2.7E-01 | 2.8E-01 |
| Hornblende (%) | |  | 4.1E-01 | 4.2E-01 | 4.7E-01 | 4.6E-01 |  |  |  |  | 4.8E-01 |  | 3.3E-01 |  |  | 4.2E-01 | 5.0E-01 |
| Ferrihydrite (%) | |  | 3.7E-01 | 2.2E-01 | 1.3E-02 | 1.3E-01 |  |  |  |  | 1.1E-01 |  | 1.4E-01 |  |  | 4.7E-02 | 3.9E-02 |
| **XRF-Sediment****** | | | | | | | | | | | | | | | | | |
| AS (mg/kg) | |  |  |  |  | 5 | 1 |  |  |  | 3 |  | 3 | 3 |  | 6 |  |
| Fe ( mg/kg) | |  |  |  |  | 23,137 | 30,185 |  |  |  | 11,273 |  | 16,133 | 16,982 |  | 24,161 |  |
| Mn (mg/kg) | |  |  |  |  | 477 | 612 |  |  |  | 178 |  | 323 | 334 |  | 406 |  |
| **ICP-MS : Water** (depth: mid-screen is 0.91 M or ( + and - 0 .455) , Well depth: 7.29, 10.85,14.34 | | | | | | | | | | | | | | | | | |
| Sample ID | |  |  |  |  | **BW07-3** |  |  |  |  | **BW10-8** |  | **BW14-3** |  |  |  |  |
| As (ug/L) | |  |  |  |  | 24.51 |  |  |  |  | 293.41 |  | 458.50 |  |  |  |  |
| Total Fe | uM |  |  |  |  | 273.65 |  |  |  |  | 294.89 |  | 354.08 |  |  |  |  |
|  | mg/L |  |  |  |  | 15.282 |  |  |  |  | 16.4681 |  | 19.7736 |  |  |  |  |
| Total S | uM |  |  |  |  | 442.5 |  |  |  |  | 111.20 |  | 125.96 |  |  |  |  |
|  | mg/L |  |  |  |  | 14.1888 |  |  |  |  | 3.5656 |  | 4.0389 |  |  |  |  |
| Total Mn | uM |  |  |  |  | 13.04 |  |  |  |  | 31.93 |  | 17.45 |  |  |  |  |
|  | mg/L |  |  |  |  | 0.7163 |  |  |  |  | 1.7542 |  | 0.9586 |  |  |  |  |
| Tritium Age | |  |  |  |  | 1.60 |  |  |  |  | 19.20 |  | 19.30 |  |  |  |  |
| **Molecular Analysis** | | | | | | | | | | | | | | | | | |
| 16S rRNA gene | |  |  |  |  | √ | √ |  |  | √ | √ |  | √ | √ |  | √ |  |
| Geobacter - 16S rRNA gene | |  |  |  |  | √ | √ |  |  | √ | √ |  | x | x |  | √ |  |
| arrA gene | |  |  |  |  | √ | x |  |  | x | x |  | x | x |  | √ |  |
| Sulphate reducers (*dsr*) | |  |  |  |  | √ | x |  |  | x | x |  | √ | x |  | √ |  |

* ‘*Blank cells’* in the table denote the absence of analysis for the respective sample, ‘√’ denotes the positive results based on PCR products of respective 16S rRNA gene, Geobacter specific 16S rRNA gene, and arrA (arsenic reductase) and dsr (sulphate reductase) functional genes and ‘x’ denotes the negative results or unamplified products .

**** The standard error for As, Fe and Mn were ≤ 1, ≤ 99 and ≤ 12, respectively.
