# Supplementary figures and images for: Microbial Community Structure and Arsenic Biogeochemistry in Two Arsenic-Impacted Aquifers in Bangladesh
Source: mBio. 2017 Nov 28;8(6):e01326-17. doi: 10.1128/mBio.01326-17 (PMC5705915; doi:10.1128/mBio.01326-17)

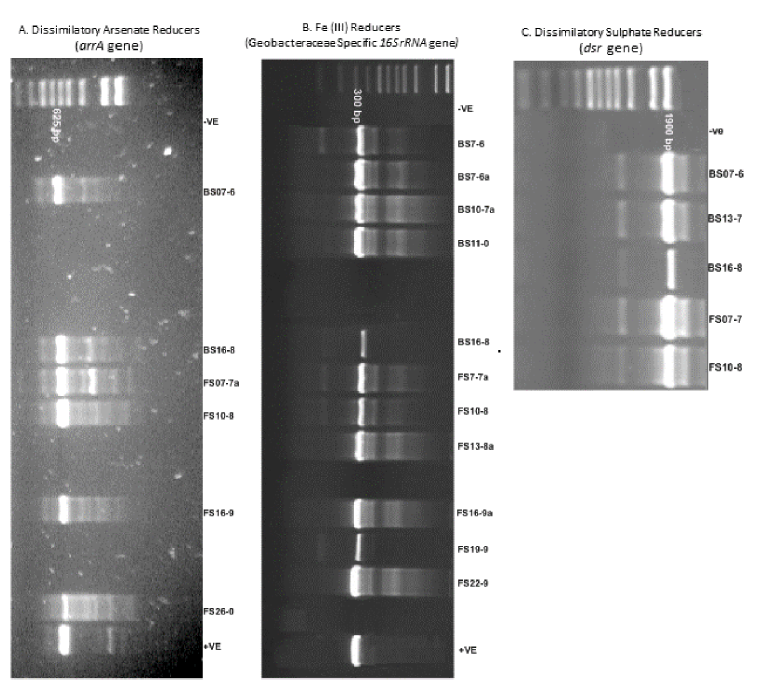

Supplement: FIG S1 [file mbo006173605sf1.tif]

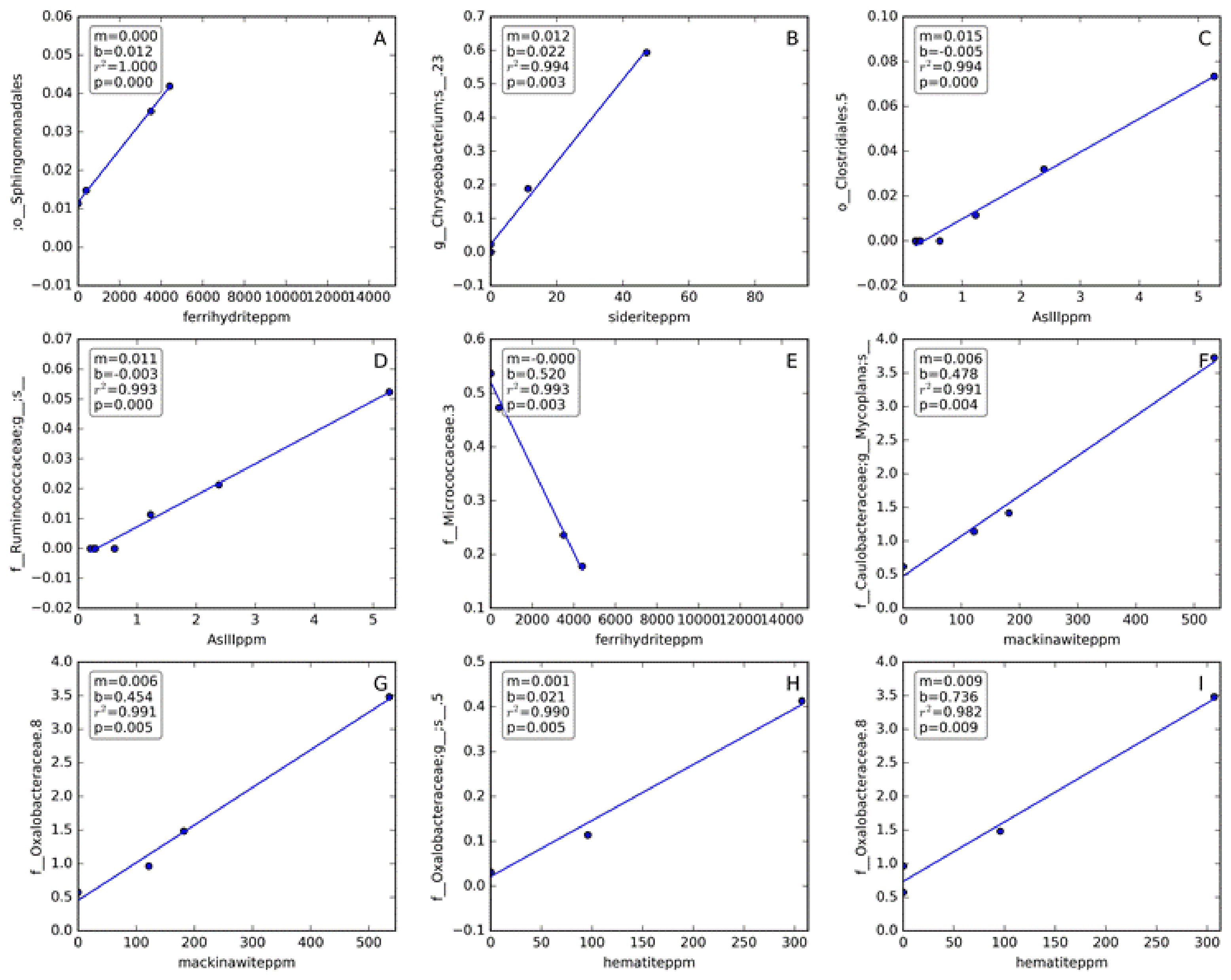

Supplement: FIG S2 [file mbo006173605sf2.tif]

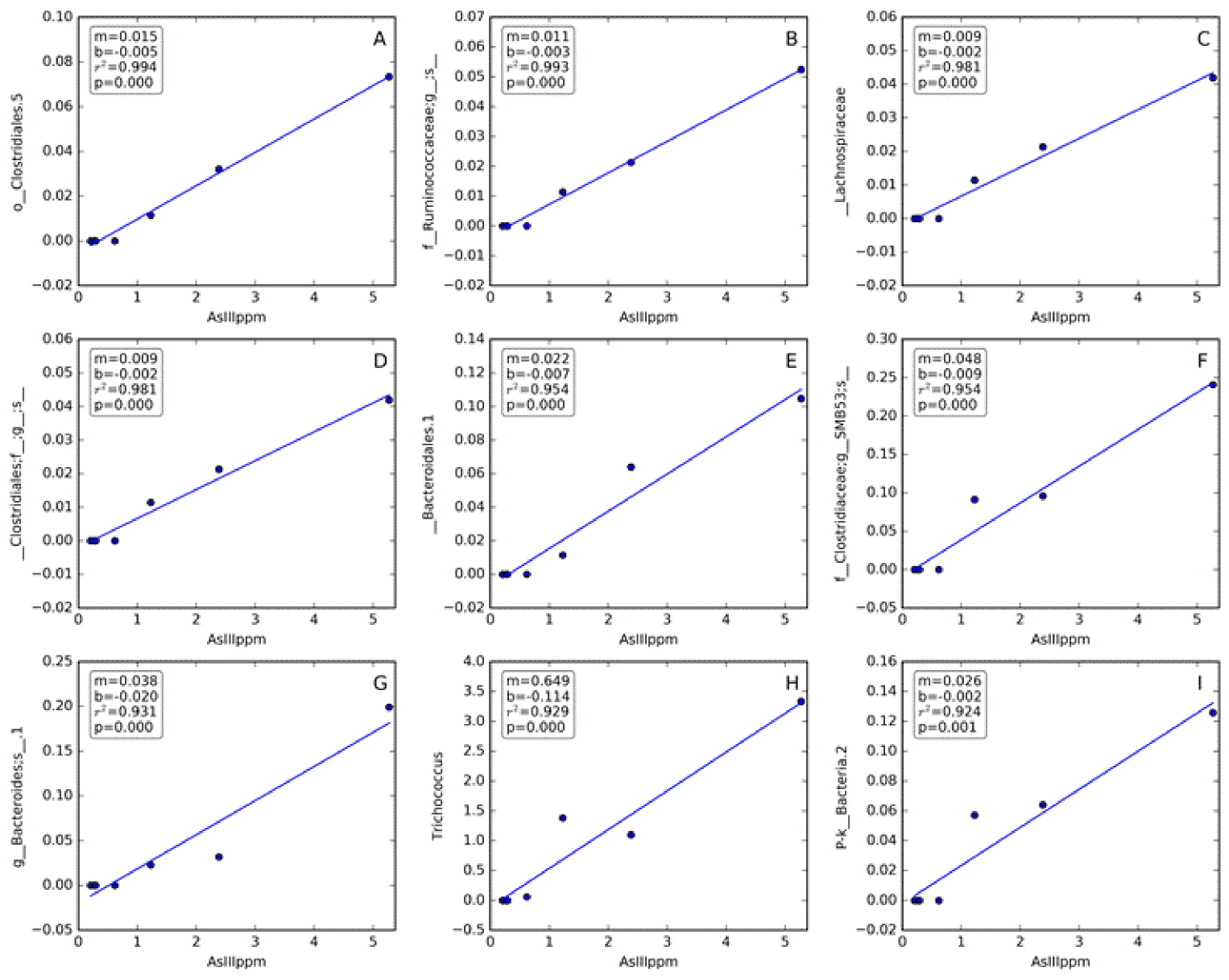

Supplement: FIG S3 [file mbo006173605sf3.tif]

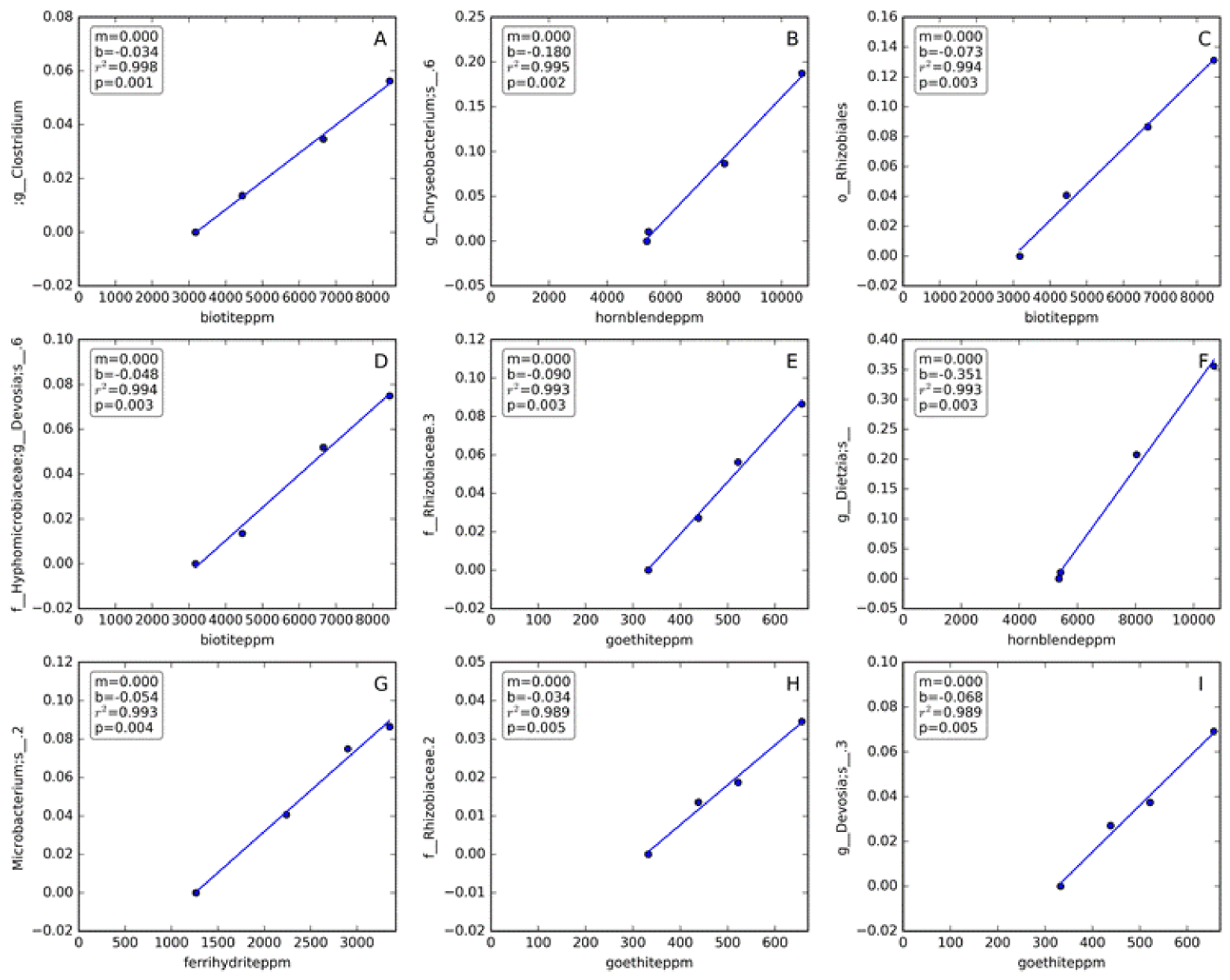

Supplement: FIG S4 [file mbo006173605sf4.tif]

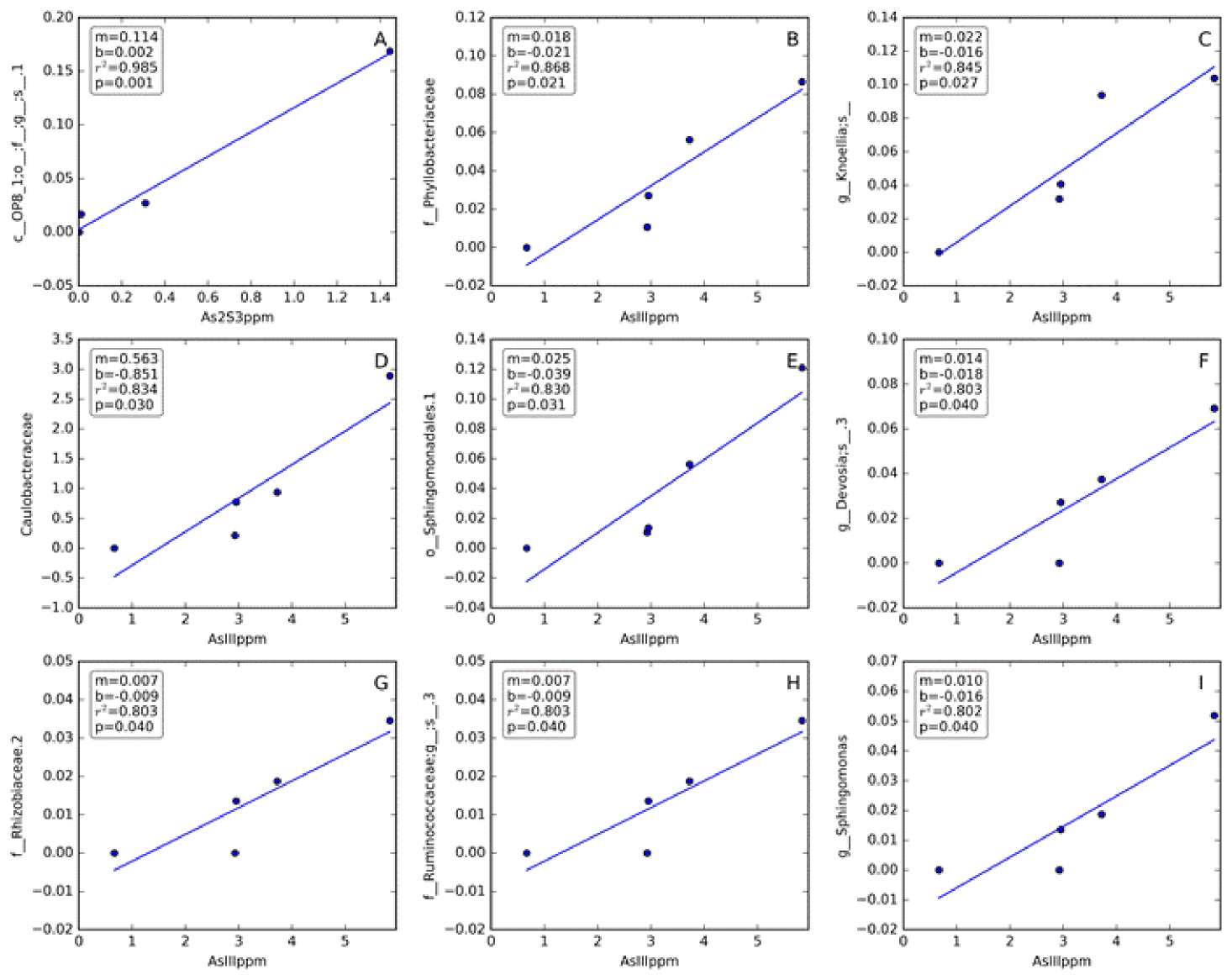

Supplement: FIG S5 [file mbo006173605sf5.tif]

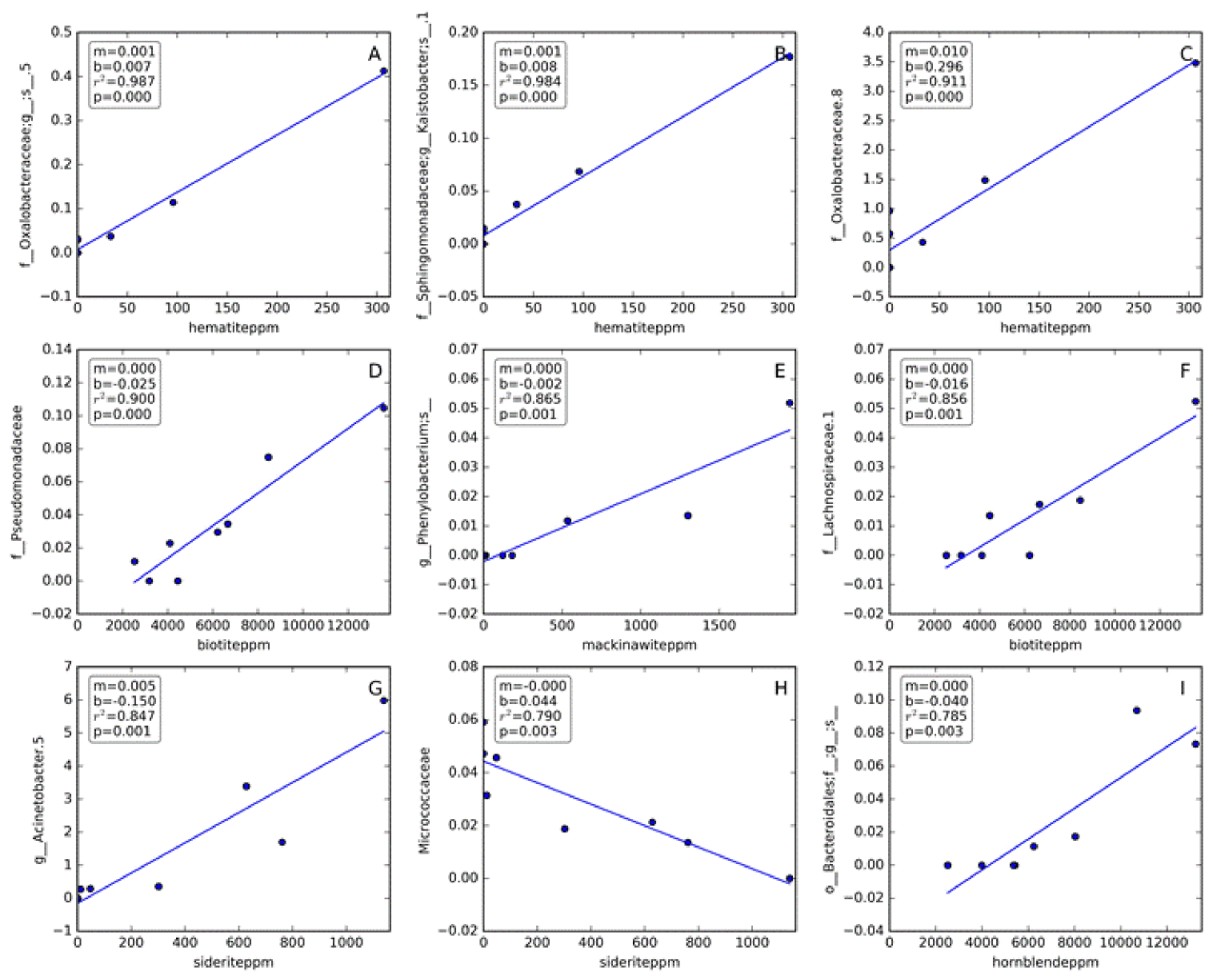

Supplement: FIG S6 [file mbo006173605sf6.tif]

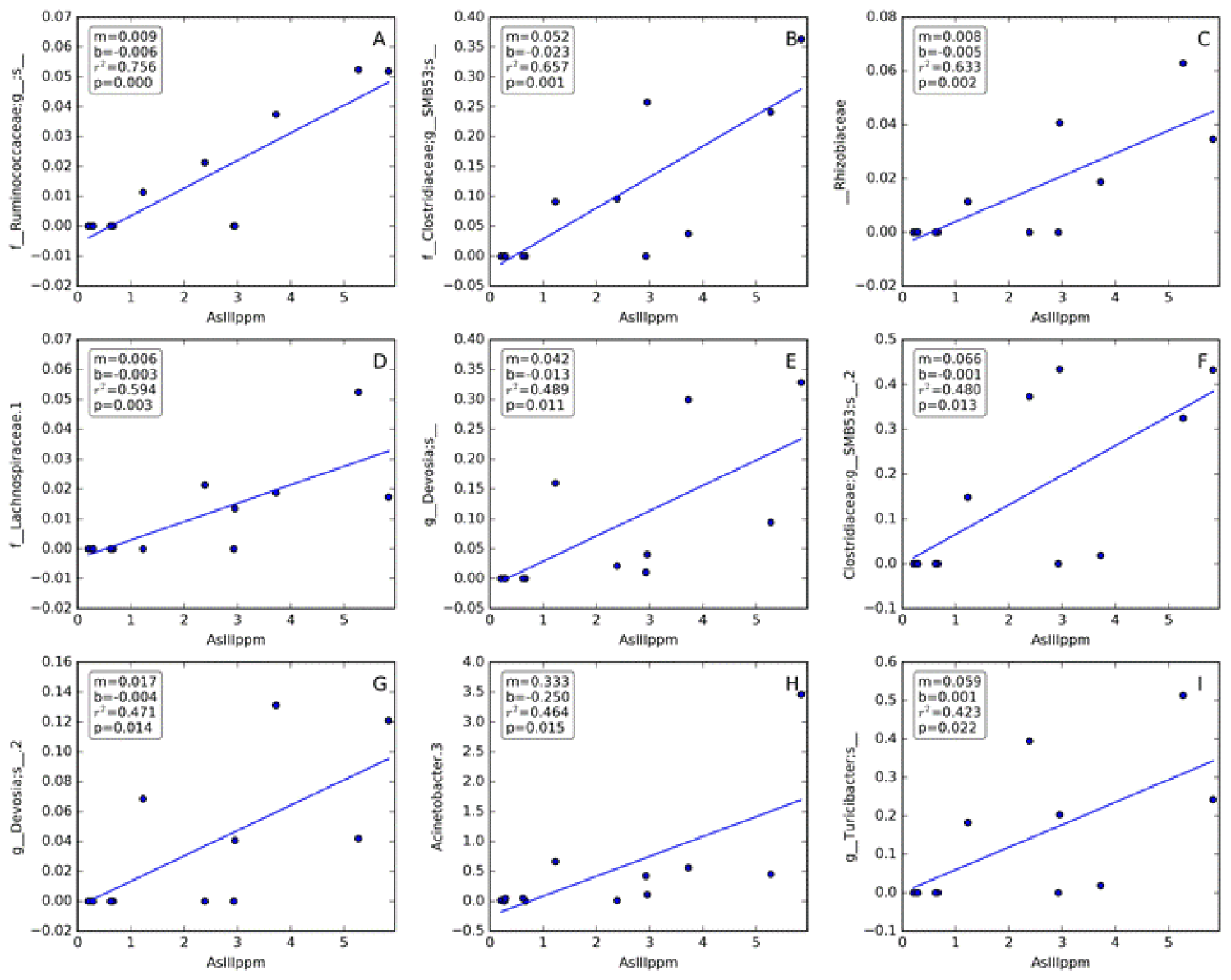

Supplement: FIG S7 [file mbo006173605sf7.tif]
