# Supplementary material for: Microbial Community Structure and Arsenic Biogeochemistry in Two Arsenic-Impacted Aquifers in Bangladesh
Source: mBio. 2017 Nov 28;8(6):e01326-17. doi: 10.1128/mBio.01326-17 (PMC5705915; doi:10.1128/mBio.01326-17)
Supplement: TABLE S3 [file mbo006173605st3.docx]

| **Table S-3.A. Percent of microbes that correlate different geochemical factors in both sold and aqueous phases (Spearman p<0.01)** | | | |  | | | | | |
| --- | --- | --- | --- | --- | --- | --- | --- | --- | --- |
|  | Site F (%) | Site B  (%) | Site B and F  (%) |  | **Table S-3.B. Number of microbial correlations in solid and aqueous phase** | | | | |
| Reflectance | 5.6 | 0.4 | 2.6 |  |  |  |  |  |  |
| AsIII ppm | 6.8 | 4.3 | 5.8 |  |  | **Solid** | **Liquid** | **Both** |  |
| AsV ppm | 5 | 0.2 | 8 |  | Site F | 86 | 5 | 9 |  |
| As_2_S_3_ ppm | 0.2 | 0.9 | 0.7 |  | Site B | 85 | 4 | 1 |  |
| Siderite ppm | 3.2 | 0.4 | 2.2 |  | Site B & F | 60 | 22 | 18 |  |
| Goethite ppm | 0.4 | 5 | 2.9 |  |  | | | | |
| Hematite ppm | 2.4 | 0.5 | 2.9 |  |  |  |  |  |  |
| Magnetite ppm | 3.2 | 0.4 | 1.8 |  | **Table S-3.C. Number of microbial correlations to 1 or more geochemical variables** | | | | |
| Mackinawite ppm | 2.4 | 0.4 | 1.1 |  |  |  |  |  |  |
| Biotite ppm | 1.4 | 10.3 | 0.7 |  |  |  | 1 variable | 2 Variables | 3 Variables |
| Hornblende ppm | 3.8 | 2.3 | 2.2 |  | Site F | Solid | 73 | 53 | 0 |
| Ferrihydrite ppm | 1.4 | 5 | 0 |  |  | Liquid | 3 | 2 | 14 |
| Aqueous As | 3.2 | 0 | 3.3 |  | Site B | Solid | 81 | 6 | 25 |
| Aqueous Fe | 3.2 | 0 | 7.7 |  |  | Liquid | 2 | 4 | 0 |
| Aqueous Sulfur | 0.2 | 0.9 | 4 |  | Site B & F | Solid | 38 | 19 | 3 |
| Aqueous Mn | 3.2 | 0.9 | 1.1 |  |  | Liquid | 18 | 13 | 0 |
